# Spontaneous closure of stage IV idiopathic full-thickness macular hole and late reopening as a lamellar macular hole: a case report

**DOI:** 10.1186/1752-1947-6-169

**Published:** 2012-06-28

**Authors:** Miriam García Fernández, Joaquín Castro Navarro

**Affiliations:** 1 Department of Opthalmology, Central University Hospital of Asturias, Oviedo, Asturias, Spain

**Keywords:** Macular hole, spontaneous closure, lamellar macular hole

## Abstract

**Introduction:**

Spontaneous closure of traumatic macular holes is described as a common event in the peer-reviewed literature. However, the spontaneous closure of stage III and IV full-thickness idiopathic macular holes has been reported in less than 15 cases in the literature, this being an extremely rare event, with their reopening being even more infrequent. We report a case of a spontaneous closure of stage IV idiopathic full-thickness macular hole and late reopening as a lamellar macular hole.

**Case presentation:**

A 67-year-old Spanish man was referred to our hospital with a complaint of decreased vision in his right eye and metamorphopsia for approximately 11 months. He did not report any trauma. Diagnosis was based on fundoscopic and optical coherence tomography. They revealed a stage IV full-thickness idiopathic macular hole and a small epiretinal membrane. Three months later the hole spontaneously closed, and two years later we appreciated its reopening as a lamellar macular hole.

**Conclusions:**

The contraction of the epiretinal membrane could have contributed to cystic spaces and their fusion, subsequently, to the formation of a lamellar macular hole. To the best of our knowledge this is the first report in the literature of a spontaneously closed full-thickness idiopathic macular hole with reopening as a partial thickness macular defect.

## Introduction

The macular hole is a full-thickness defect of retinal tissue involving the anatomic fovea. There exists controversy regarding the pathogenesis, prognosis and treatment of this condition.

Spontaneous closure of traumatic macular holes is described as a common event in the peer-reviewed literature. However, the spontaneous closure of stage III and IV idiopathic macular holes has been reported in less than 15 cases in the literature, this being an extremely rare event [1-8]. Furthermore, there are only two reports about spontaneous reopening of a spontaneously closed macular hole [9,10].

However, to the best of our knowledge, there are no papers describing the reopening as a lamellar macular hole after a long-standing spontaneous closure of a full-thickness macular hole (FTMH).

## Case presentation

A 67-year-old Spanish man with unremarkable ocular and systemic history, except for a depressive syndrome, was referred to our hospital. He presented with decreased vision in his right eye and a history of metamorphopsia for approximately 11 months. He did not report any trauma. His best-corrected visual acuity (BCVA) was 0.5 in the right eye and 1.0 in the left eye.

**Figure 1:**
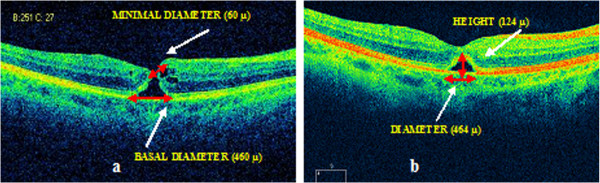
**Cirrus optical coherence tomography (OCT) scan. (a)** Scan at first visit, showing a stage IV full-thickness idiopathic macular holes (FTMH). The basal and minimal macular hole diameters were 460 μ and 60 μ, respectively. Clear irregularity of the junction of inner and outer segments of photoreceptors (IS/OS) and a small epiretinal membrane (ERM) can be observed. **(b)** Scan at the third month follow-up visit, revealing a closed macular hole, and a cystic space that represents the bridging effect. A disruption of the photoreceptor layer can also be appreciated. The basal diameter of the cystic space is 464 μ, and the height of the elevation 124 μ.

Anterior segment examination revealed a bilateral nuclear sclerosis with no further abnormalities. Fundoscopy revealed an image of a full-thickness macular hole. Optical coherence tomography (OCT) examination (Figure 1a) showed a stage IV FTMH according to its size (more than 400 μ) and according to the posterior vitreous detachment, the latter only visible on fundoscopy examination.

We also appreciated some cystic spaces on both edges of the hole, and a small epiretinal membrane (ERM). We observed clear irregularity of the junction of inner and outer segments of photoreceptors (IS/OS).

Three months later, his BCVA had not improved. OCT scans revealed a closed macular hole. We observed an elevation of the photoreceptor layer and of the external limiting membrane (ELM) over a cystic space, and a defect in the continuity of the photoreceptor layer (Figure 1b).

**Figure 2:**
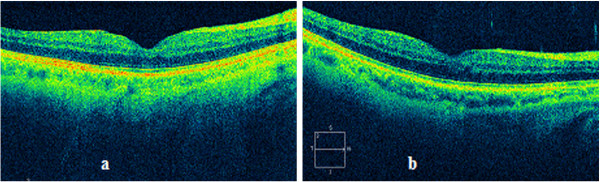
**Cirrus optical coherence tomography (OCT) scan.** Scans from the seventh-month **(a)** and 13th-month **(b)** follow-up visits, respectively, showing the disappearance of the cystic space, and a normal foveal contour, although a small interruption in the line corresponding to the photoreceptors (IS/OS) and in the external limiting membrane (ELM) can be appreciated.

Seven months later, his BCVA remained the same. An OCT examination showed the disappearance of the cystic space, and a normal foveal contour (Figure 2a).

At 13 months later, his BCVA improved to 0.7. An OCT scan showed no changes (Figure 2b).

**Figure 3:**
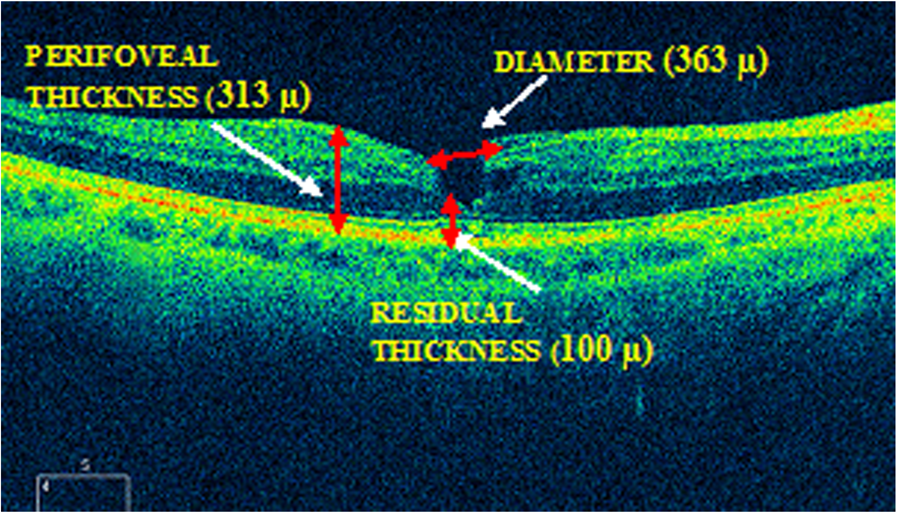
**Cirrus optical coherence tomography (OCT) scan.** Two years later, we observed a lamellar macular hole, according to the Haouchine criteria. However, we appreciated damage to the photoreceptors, which does not usually appear in lamellar macular holes.

Two years later, his BCVA remained unchanged but on OCT scan we surprisingly observed a lamellar macular hole (Figure 3), according to the Haouchine [11] criteria: irregular thinning of foveal base, break in the inner fovea, intra-retinal split (dehiscence of the inner foveal retina from the outer retina), normal perifoveal retinal thickness and absence of a full-thickness foveal defect.

## Discussion

The spontaneous closure of idiopathic macular holes is less prevalent and less well understood than closure of traumatic macular holes [4].

Several mechanisms for the spontaneous closure of idiopathic macular holes have been described: a complete detachment of the posterior hyaloids, leading to a reduction in antero-posterior tractional forces [7]; the bridging effect of retinal tissue and glial cell proliferation across the hole [5,8,9]; and the formation of a contractile ERM, which provokes shrinkage of the hole, and the cells proliferation at its base [12].

There are reported cases of spontaneous closure of FTMH in one eye after vitreoretinal surgery for FTMH in the other eye. In these cases, the prone position and gravitational forces involved can determine the complete detachment of the posterior vitreous, releasing the forces that contribute to FTMH progression [2,3].

In our patient, we think that it is likely a vitreomacular traction started the formation of the FTMH. After that, a vitreous posterior detachment occurred, and, as the antero-posterior tractional forces released, the macular hole began to close.

The first time we observed our patient, the opening diameter of the hole was over six times smaller than the basal diameter; this may have been due to the fact that the restoration had already begun.

Three months later, the process of closure continued, and the bridging effect started. Several authors consider this as the first step in macular hole closure [5,8,9].

Six months later (three months after the observation of the bridging effect) we were able to observe the complete closure of the macular hole.

Despite this, our patient’s BCVA did not improve to 1.0. It is likely that the disruption in the photoreceptor layer had resulted in worse visual acuity.

Interestingly, three months is also the time required to the definitive closure since the starting of the bridging effect, in other similar report in the literature [8].

There are two distinct features of our patient. Firstly, the relatively old age for the spontaneous closure, and secondly, which is more relevant, the formation of a lamellar macular hole one year after the closure (and two years since the development) of the FTMH, this being, to the best of our knowledge, the first case reported in the peer-reviewed literature.

Several factors have been described for the reopening of full-thickness macular holes after vitrectomy: cataract extraction (although controversial) [13], neodymium: yttrium aluminum garnet (YAG) capsulotomy [14], tangential contraction of an ERM [15], and so on. However, the pathogenesis of spontaneous reopening is still controversial.

In the case of the tangential contraction of an ERM, the mechanism is the formation of intra-retinal cysts and their posterior rupture and fusion. This cystoid macular edema (CME) can also be secondary to age-related macular degeneration, diabetic retinopathy, inflammatory and vascular diseases, and so on.

These factors could be also valid for lamellar macular holes, considering the fact that lamellar holes are described as an abortive process in the development of FTMH formation.

## Conclusions

To the best of our knowledge, this is the first case in the peer-reviewed literature that describes the late evolution of a spontaneously closed full-thickness macular hole to a lamellar macular hole.

In our patient, the mechanism of formation of a lamellar macular hole after spontaneous closure of a stage III to IV idiopathic macular hole remained unanswered.

We postulate that, considering the absence of any surgery, inflammatory or vascular diseases during the follow-up, the antero-posterior traction of the small epiretinal membrane, and consequently, the formation of intra-retinal cysts, with posterior rupture and fusion, might have played a role in the development of the lamellar macular defect.

In those cases of spontaneously closed FTMH with associated ERM, we may consider periodic observation as the progression of the ERM can contribute to the formation of a lamellar macular hole, which, although infrequently, could progress to a new FTMH.

## Consent

Written consent was obtained from the patient for publication of this case report and any accompanying images. A copy of the written consent is available for review by the Editor-in-Chief of this journal.

## Competing interests

The authors declare that they have no competing interests.

## Authors’ contributions

MGF was involved in data acquisition and manuscript drafting. JCN was involved in data interpretation and revised the report critically for important intellectual content. All authors read and approved the final manuscript.
